# Polycystic Ovarian Syndrome: A Complex Disease with a Genetics Approach

**DOI:** 10.3390/biomedicines10030540

**Published:** 2022-02-24

**Authors:** Himani Nautiyal, Syed Sarim Imam, Sultan Alshehri, Mohammed M. Ghoneim, Muhammad Afzal, Sami I. Alzarea, Emine Güven, Fahad A. Al-Abbasi, Imran Kazmi

**Affiliations:** 1 Siddhartha Institute of Pharmacy, Near IT-Park, Sahastradhara Road, Dehradun 248001, India; himanibpharma2011@gmail.com; 2 Department of Pharmaceutics, College of Pharmacy, King Saud University, Riyadh 11451, Saudi Arabia; simam@ksu.edu.sa (S.S.I.); salshehri1@ksu.edu.sa (S.A.); 3 Department of Pharmacy Practice, College of Pharmacy, AlMaarefa University, Ad Diriyah 13713, Saudi Arabia; mghoneim@mcst.edu.sa; 4 Department of Pharmacology, College of Pharmacy, Jouf University, Sakaka 72341, Saudi Arabia; samisz@ju.edu.sa; 5 Biomedical Engineering Department, Faculty of Engineering, Düzce University, Düzce 81620, Turkey; emine.guven@duzce.edu.tr; 6 Department of Biochemistry, Faculty of Science, King Abdulaziz University, Jeddah 21589, Saudi Arabia; fabbasi@kau.edu.sa

**Keywords:** polycystic, biochemical, hyperandrogenism, multigene, ovulatory

## Abstract

Polycystic ovarian syndrome (PCOS) is a complex endocrine disorder affecting females in their reproductive age. The early diagnosis of PCOS is complicated and complex due to overlapping symptoms of this disease. The most accepted diagnostic approach today is the Rotterdam Consensus (2003), which supports the positive diagnosis of PCOS when patients present two out of the following three symptoms: biochemical and clinical signs of hyperandrogenism, oligo, and anovulation, also polycystic ovarian morphology on sonography. Genetic variance, epigenetic changes, and disturbed lifestyle lead to the development of pathophysiological disturbances, which include hyperandrogenism, insulin resistance, and chronic inflammation in PCOS females. At the molecular level, different proteins and molecular and signaling pathways are involved in disease progression, which leads to the failure of a single genetic diagnostic approach. The genetic approach to elucidate the mechanism of pathogenesis of PCOS was recently developed, whereby four phenotypic variances of PCOS categorize PCOS patients into classic, ovulatory, and non-hyperandrogenic types. Genetic studies help to identify the root cause for the development of this PCOS. PCOS genetic inheritance is autosomal dominant but the latest investigations revealed it as a multigene origin disease. Different genetic loci and specific genes have been identified so far as being associated with this disease. Genome-wide association studies (GWAS) and related genetic studies have changed the scenario for the diagnosis and treatment of this reproductive and metabolic condition known as PCOS. This review article briefly discusses different genes associated directly or indirectly with disease development and progression.

## 1. Introduction

Polycystic ovarian syndrome (PCOS) is a multifactorial disorder affecting females in their reproductive ages. This condition is diagnosed in females with hyperandrogenism, oligomenorrhea, amenorrhea, acne, hirsutism, insulin resistance, obesity, and infertility [1,2]. It is well-documented that more than 40% of female infertility is associated with PCOS [3]. Additionally, females suffering from PCOS are more prone to endometrial cancer [1]. Other metabolic conditions such as diabetes mellitus, hepatic steatosis, dyslipidemia, and cardiac complications are also present in PCOS patients [4]. Various reported studies have shown that physiological alterations are complimented with various psychological symptoms in patients, such as poor self-esteem, depression, negative body image, and decreased quality of life [5].

The genetic development of PCOS depends on individual genes, gene–gene interactions, and altered genes’ environmental conditions. It is important to identify the variant key gene that changes its expression and sequence to modify protein function in order to determine the genetic makeup of PCOS [6]. Epigenetic alterations at the tissue level are responsible for cells changing their phenotype in response to the changed environment [7]. The proteome profile in pathophysiological tissues describes the changes in the cellular proteome [8]. The molecular pathophysiology of PCOS depends on genetic and epigenetic patterns affecting synthesized proteins, which further contribute to PCOS biomarkers.

## 2. Epidemiologic Profile of PCOS

Recently, different diagnostic criteria have been adopted for the determination of PCOS in a patient, including hyperandrogenism, dysfunctional ovaries, and polycystic ovaries morphology (PCOM) and its combination. Initially, National Institute of Health (NIH), in 1990, proposed hyperandrogenism and chronic anovulation as important diagnostic symptoms for PCOS [8]. Later on, in 2003, PCOM was also included as a diagnostic criterion by Rotterdam Consensus [9]. In 2006, Androgen Excess Society included hyperandrogenism as an essential symptom for diagnosis [10].

Current recommendations suggest Rotterdam consensus for PCOS diagnosis, including four phenotypes [11] (Table 1).

PCOS prevalence depends on the number of criteria followed for the diagnosis. In a study, a 4% prevalence rate was found in both Caucasian and Black races [12]. From 1999 through 2016, in a multicentric study conducted on 1089 patients of PCOS from different ethnicity and race, a significant impact of environmental and racial factors on metabolic syndrome produced in PCOS patients was reported [13]. In a study conducted on Spanish Caucasian women, it was found that the PCOS prevalence rate was at 6.5% [14]. In a combined study conducted at Oxford University and a private healthcare setting, the prevalence rate was also found to be 6.8% [15].

A study conducted in females of reproductive age in China showed a prevalence rate of 5.6%, which was quite similar to other reported studies [15,16]. In Indian women of reproductive age, PCOS was found to be more prevalent by 9.13% [15,17]. Another study in India showed 8.8% prevalence among adolescent girls [18]. PCOS was found to be more prevalent in South Asian females when compared to Caucasian females. Based on 2003 Rotterdam criteria, higher prevalence rate was found in Pakistani females (almost 50%) in a study by Akram and Roohi in 2015 [15,19]. Other studies from Pakistan reported almost 40% prevalence in infertile females visiting medical health centers in Pakistan [15,20].

## 3. Window of Susceptibility

PCOS can affect women’s health at any stage of life. This phenotypic difference requires personalized diagnosis and treatment approaches among different ages. Diagnosis of PCOS in adolescence is more difficult due to similar physiological patterns in puberty and PCOS. As a person ages, this syndrome changes into a reproductive disease which further evolves into a metabolic disorder [21].

### 3.1. PCOS in Adolescence

The normal pubertal cycle may also indicate an irregular and anovulatory menstrual cycle, which leads to difficulty in diagnosis of PCOS among adolescent girls. The age at menarche is associated with the onset of the regular ovulatory cycle [22]. Half of the menstrual cycles in the first 4.5 years of the menarche will be ovulatory for girls who reach their menarche after the age of 13. If a girl is an oligo-amenorrheic at the age of 15, she will show such symptoms in her adult life too [23]. A study report about international evidence-based guidelines suggests that after menarche (in less than 2 years), if girls show signs related to PCOS, it could be regarded as “at high risk for PCOS”. Such girls should be longitudinally followed up and re-evaluated for eight years after menarche [24].

### 3.2. PCOS in Reproductive Age

The anovulatory subfertility is featured in almost 70% of PCOS patients in their reproductive ages [25]. A study reports 15-fold subfertility in PCOS patients when compared with independent BMI controls. Additionally, female hormones treatment was reported more often with PCOS patients (62%) when compared with non-PCOS patients (33%). IVF treatment was found to be similar in PCOS and non-PCOS females [26]. Pregnancy and delivery complications have been reported in various meta-analysis studies. Gestational diabetes, pre-eclampsia, cesarean section, and gestational hypertension have also been reported [21,27,28]. Obesity is associated with insulin resistance and hyperinsulinemia, which are further responsible for hyper adipogenesis and low lipolysis. Theca cells sensitivity is also aggravated for the luteinizing hormone, which leads to functional ovarian hyperandrogenism. These findings suggest that PCOS phenotype (reproductive and metabolic) seems to be potentiated by obesity [21,29].

### 3.3. PCOS in Menopausal Age

Menopause is a life stage that occurs at almost 51 years of age. There are limited data available in terms of longitudinal natural history studies, which leads to confusion among health care professionals in screening recommendations for the disease progression and assessment in long-term health risk [24]. PCOS patients have similar aging symptoms as in the normal physiology, which is the loss of follicles and disappearance of polycystic ovarian morphology later on [30]. PCOS patients also show a similar shortening of their menstrual cycle length, and these females turn to eumenorrheic subsequently. Different studies also reported that menopause occurs later in PCOS females’ lives, and these females might have a regular ovulatory cycle at the end of their reproductive years. The selective menopause-postponing genetic variants enrichment in PCOS females is responsible for the delay in the occurrence of menopause [21,31].

## 4. Clinical Pattern of Disease

**Figure 1:**
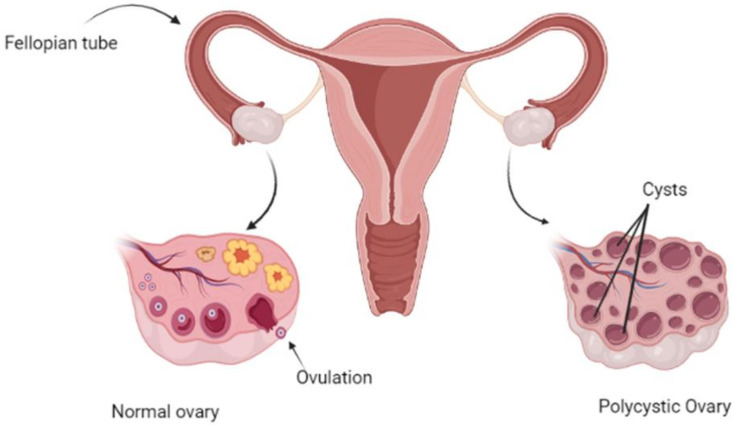
Diagrammatic Representation of Polycystic Ovary Syndrome.

Infertility caused by PCOS produces a social imbalance and burdens females affected with this disorder. The elevated level of androgen leads to subcellular aberration in ovarian theca cells. Intrinsic activation of theca cell steroidogenesis leads to androgen excess despite the absence of tropic factors. This intrinsic activation also influences granulosa cell to increase the level of the anti-Mullerian hormone (AMH) in PCOS patient [32,33]. Various studies have reported an increased number of antral and pre-antral follicle cells in PCOS patients, a defective apoptotic activity in the mature follicular cells that results in the elevation of an abnormally developed follicle number, which is further responsible for the availability of ovarian cysts in patients [34,35] (Figure 1).

**Figure 2:**
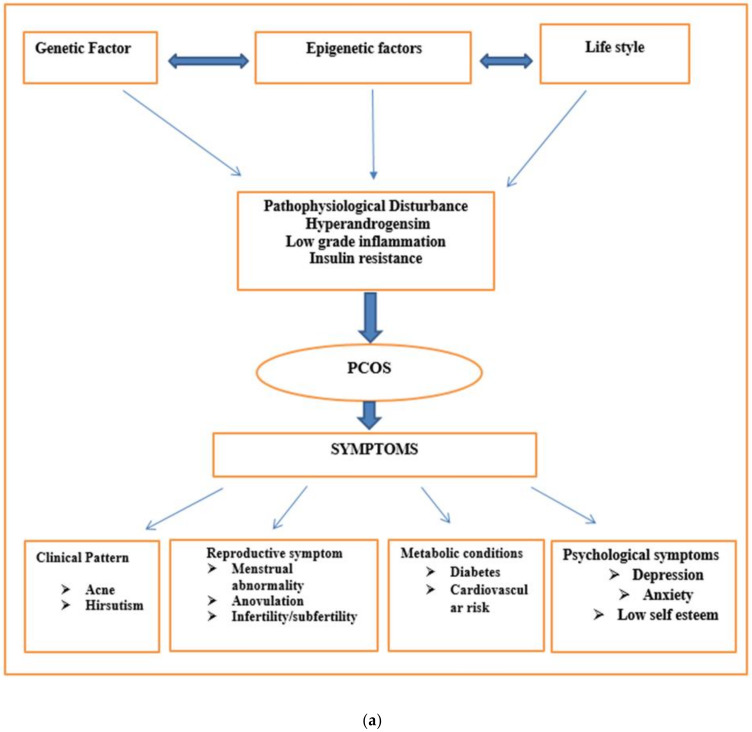

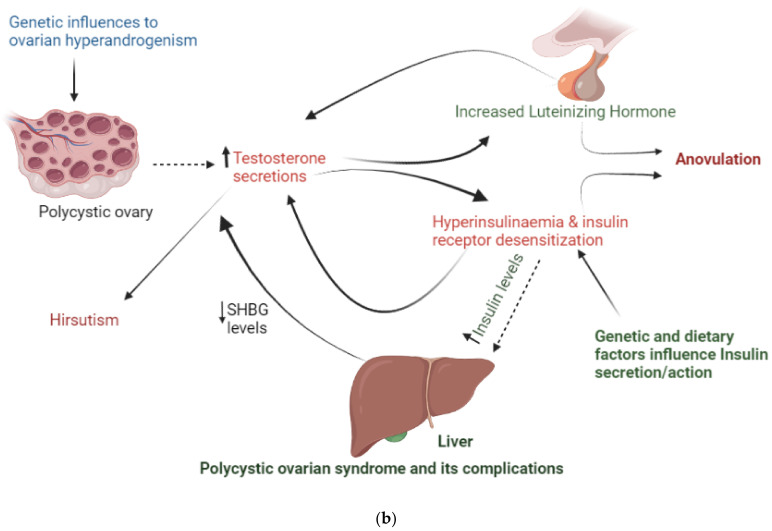
(**a**) Pathophysiologic features of polycystic ovary syndrome. PCOS is a multifactorial disorder that develops due to the combined effects of impaired genetic, epigenetic, and lifestyle factors. All these factors lead to disturbed pathophysiology (insulin resistance, hyperandrogenism, and low grade inflammation, which further result in complex symptoms of PCOS (Clinical, Reproductive, Metabolic, and Psychological). (**b**) Genetic influences and complications of polycystic ovarian syndrome. Different genetic polymorphism influences the pathophysiology of PCOS. Genetic influence alters different hormonal levels—androgen, insulin, AMH, SHBG, LH, FSH, testosterone, and different other hormonal levels, which further contribute to disease progression.

The insulin signaling defect is an important clinical marker (insulin resistance) of PCOS, which is independent of obesity. The altered insulin gene expression pathway and the glyco-oxidative pathway are also involved in pathogenies of PCOS [36,37,38]. High oxidative stress results in impaired insulin activity, which further results in hyperandrogenism (Figure 2).

## 5. Phenotypic Classification of PCOS

Four types of observable characteristics have been found in patients in different studies: Phenotype A, Phenotype B, Phenotype C, and Phenotype D.

### 5.1. Classic Phenotype

Women with PCOS Phenotype A and B are termed classic PCOS patients. These patients show significant symptoms associated with menstrual dysfunction, hyperinsulinemia, insulin resistance, and increased risk of metabolic syndrome. Different reported studies found that these patients are more prone to obesity and dyslipidemia [39,40]. Sahmay and his associates found increased serum levels of anti-Mullerian hormone in classic PCOS patients as compared to other phenotypes [41].

### 5.2. Ovulatory PCOS

Phenotype C patients are considered to be ovulatory PCOS patients. It has been found in a cohort study that females with a higher socioeconomic status are more prone to such conditions. Due to their eating habits, the insulin level and fat distribution are disturbed in their body which results in ovulatory imbalance among them [42]. A slight elevation of insulin, androgen, and lipid profile is a common indication of Phenotype C. Ovulatory PCOS patients show more profound metabolic symptoms as compared to classic PCOS [43].

### 5.3. Non-Hyperandrogenic PCOS

Non-hyperandrogenic PCOS patients are considered as Phenotype D. In this phenotype, patients show normal androgen with elevated endocrine levels and defective metabolic conditions [44,45]. As compared to classic PCOS, a lower (Luteinizing Hormone/Folicle stimulating Hormone) LH/FSH ratio can be found in this phenotype, with altered levels of thyroid hormone (T3, T4) and sex hormone-binding globulin. Phenotype D patients have regular menstruation with intermittent irregularities [46,47].

Different studies reported the frequency of phenotypic occurrence in a patient population, as Phenotype A is found in 44–65%, Phenotype B in 8–33%, Phenotype C in 3–29%, and Phenotype D in 0–23% [43,48]. A separate study by Moghetti and his colleagues reported the PCOS phenotype among patient population as 70% classic, 15% as ovulatory phenotype, and 15% as non-hyperandrogenic [46]. In studying the insulin resistance among PCOS patients, Moghetti et al. concluded that almost 70% of females had insulin resistance; however, the percentage varied across different PCOS phenotypes, with 80% of females in classic, 65% of females in ovulatory PCOS, and 38% of females in non-hyperandrogenic PCOS [46] (Table 2). The study reported by Lizneva et al. (2016) indicates a 40–45% prevalence rate of classic PCOS, 35% for ovulatory PCOS, and 20% for non-hyperandrogenic PCOS in an unselected population [39] (Table 2).

The classification of PCOS depends on the following diagnostic criteria:

- Hyperandrogenism, oligo anovulation, various polycystic ovaries morphology (through ultrasound)
- Hyperandrogenism, oligo anovulation, normal ovaries morphology (through ultrasound)
- Hyperandrogenism, normal menstruation cycle, various polycystic ovaries morphology (through ultrasound)
- Normal androgen, oligo anovulation, and polycystic ovaries morphology (through ultrasound)

## 6. PCOS Pathogenesis

### 6.1. Hyperandrogenism

PCOS is considered to be an intrinsic disorder of the ovaries, whereby hypersynthesis of androgen occurs due to certain genetic defects. Androgen is synthesized from ovarian theca cells in response to the luteinizing hormone. The rate-limiting step of steroidal hormone synthesis, 17α hydroxylase and 17, 20 lyase activities occurs in the theca cells, which express the CYP17A1 gene encoding the P450c17 enzyme responsible for the above reaction [49,50]. Both intra and extra ovarian mechanisms are involved in androgen production. Increased levels of the luteinizing hormone downregulates the LH receptors, which further decreases CYP17A1 expression and further negatively affects androgen production. An autocrine and paracrine negative feedback mechanism through estrogen and androgen inhibits 17α hydroxylase and 17, 20 lyase activity. LH receptor upregulation and P450c17 are potentiated by insulin and insulin-like growth factor (IGFs) [51]. As per various in vitro studies, it was found that PCOS theca cells produce more androgen compared to normal control. This increased androgen synthesis is due to 17α hydroxylase and 17, 20 lyase hyperactivity. Cholesterol side-chain enzymes action depicts the intrinsic theca cell defects [52]. Overall, 20–30% of PCOS women have higher ovarian androgen along with adrenal androgen, dehydroepiandrosterone (DHEA), and dehydroepiandrosterone sulfate (DHEAS) [53].

Ovarian theca cell enzymes resemble adrenal zona reticularis, which favors the formation of DHEA and DHEAS (sulfated by sulfotransferase 2A1 [SULT2A1]). DHEAS is a potent inert terminal product, which is again converted to DHEA and metabolized in other androgens. Due to genetic variation of SULT2A1, PCOS patients vary in DHEA/DHEAS ratio [54]. Adrenal hyperandrogenism, indicative in PCOS patients, does not depend on hypothalamus pituitary adrenal gland axes’ mediated response but depends on hyperresponsiveness toward androgen synthesis [53]. Increased P450c17 enzyme activity is responsible for this hyperandrogenism. A study reported (Draper et al., 2004) that genetic and epigenetic variation affects PCOS hyperandrogenism. Cortisone reductase deficiency is an example of a genetic cause of adrenal PCOS. In 11 β hydroxysteroid dehydrogenase type 1 deficiency, cortisone is not converted to cortisol, which further elevates the ACTH level and leads to hyper-androgen production [55].

### 6.2. Ovarian Follicular Dysfunction

The ovarian follicular recruitment process is independent of gonadal hormones. Oocytes secrete different inhibitory transcription factors serine/threonine kinase (LKB1, STK11, and BMP4) and proapoptotic factors FOX (forkhead box) and perform epithelial–mesenchymal interaction to control follicular growth in a quiescence state [56]. Follicular growth factors are involved in the regulation and control of ovarian folliculogenesis. These follicular growth factors (BMP9, GDF9, BMP6, and BMP15) work in synchronicity and synergistically to regulate the growth and development of follicular cells [51]. The TGF-β superfamily (BMP), inhibin B, cytokines, and microRNA also play important roles in this process [57]. Ovarian dysgenesis-mediated ovarian failure is the result of a mutation in BMP15 [58,59,60]. Meikeli and his colleagues found a positive correlation between FOX3 expression and activation in granulosa cells with apoptosis, which describes the FOX transcription factor as a potential target for PCOS [61].

Another important folliculogenesis modulator is AMH, which is produced by granulosa cells of small growing follicles. A pre-antral and small antral follicle detects the highest expression of ≤4 mm. A grown follicle (>8 mm) loses AMH expression and becomes more sensitive to FSH action. In normal ovaries, it further determines follicular growth, estrogen production, the selection of dominant follicle, and resulting ovulation [56].

AMH is involved in the inhibition of the initial primary follicular recruitment from the primordial follicle pool (FSH independent), which also inhibits follicular maturation and a selection of the dominant follicle (FSH dependent) [57]. It has been found in different studies that the AMH level is significantly high in PCOS women, where follicles are arrested in the pre-antral and antral states. In both stages, AMH production is high [31,56,58]. The granulosa cells of anovulatory PCOS patients have 75 times greater production of AMH per cell [31]. As per this study, high AMH concentration was found to be a causative factor for anovulation in PCOS patients along with the consequence of increased granulosa cell mass [31,56].

### 6.3. Neuroendocrine Imbalance

A gonadotropic hormone is associated with ovarian androgen synthesis, either directly or indirectly. Higher pulse frequency of LH is an important indicator of PCOS. This high pulse is due to negative feedback of progesterone in response to GnRH pulse resistance. However, a neuroendocrine axis imbalance is secondary to hyperandrogenism. Since imbalanced LH pulse frequency is an inconstant feature in PCOS patients, it is not classified as a primary feature in PCOS (Figure 2a).

### 6.4. Insulin Resistance

Defective insulin autophosphorylation and reduced insulin receptor binding lead to insulin resistance (Figure 2b).

## 7. Inheritance in PCOS

A common pathway is associated with the genetic basis of PCOS within the family and between the families. Patients from the same family have a different genetic susceptibility for PCOS [60]. Female siblings have menstrual and hyperandrogenic features similar to their mothers, while male siblings show hyperandrogenic symptoms as early in the development of baldness [61]. As per initial genetic investigational discoveries, PCOS inheritance is autosomal dominant but further investigations revealed that it is a multigene origin disease.

Due to insufficient and inconsistent diagnostic criteria, researchers continue to face different investigational challenges. Genome-wide association study (GWAS) and candidate gene studies are two complementary gene studies. GWAS studies are involved in finding some association between genetic polymorphism and the disease trait while a predefined hypothesis is unavailable; it discusses the genetic variant role in pathophysiology. The genetic loci (region) associated with disease traits are found in these studies. These genetic regions are either directly involved in the genetic function if they are near or in the gene, or if there are involved genetic upregulations or downregulations. Various GWAS studies (Chinese, European and Korean) have been performed to detect around 19 different genetic loci associated with PCOS [62,63,64,65,66,67,68].

Candidate gene studies are used to validate GWAS study findings; these studies identify single nucleotide polymorphism (SNP) contributing to PCOS pathophysiology [63,69]. These studies are effective genetic-variant-detection methods, but they also have some limitations, including sample size, diagnostic criteria, and participant sources which fluctuate statistically to cause variation in results [70,71,72,73,74,75,76].

## 8. Genetic Heritability Reported in GWAS Studies

GWAS have reported various biological pathways involved in PCOS pathophysiology. Isoform 1A (DENND1A), which is differentially expressed in normal and neoplastic development, has been found to be a potential risk factor, which encodes a protein called connecdenn-1 associated with clathrin-coated pits residing in cell surface receptors. Theca cell’s nucleus and cytoplasm contain DENND1A protein [51]. This protein contains two important transcript variants (DENND1A.V1 and DENND1A.V2), which further encode 1009-aa protein with proline-rich domain C terminal (DENND1A.V1) and truncated 559-aa protein containing DENN and clathrin domain. The protein, which lacks proline-rich domain but includes C terminal 33-aa sequence, is not available in connecdenn-1 [77]. A study reported (DENND1A.V2) overexpression in PCOS theca cells. Increased CYP17A1 and CYP11A1 expression and hyper androgen synthesis were reported in normal theca cells, where (DENND1A.V2) is forced to overexpress. Additionally, DENND1A expression was recently found in adrenal zona reticularis [78]. All these studies identified DENND1A.V2 as a potential intrinsic factor involved in the steroidogenic pathway in PCOS. The luteinizing hormone/choriogonadotropin receptor (LHCR) gene is G-protein coupled receptor (GPCR) expressed in preovulatory follicular granulosa cells. Increased LHCR expression in granulosa cells of follicles leads to response to LH peak, which further helps in ovulation. Abnormal mutations in this gene results in amenorrhea, oligomenorrhea, infertility, and also hyperandrogenism [79,80,81]. The follicular stimulating hormone receptor (FSHR) gene is responsible for ovarian responses toward FSH. The abnormal mutation leads to follicular arrest in the antral state. A polymorphic change in this gene leads to the hyperconcentration of FSH and decreased susceptibility toward exogenously administered gonadotropins and clomiphene citrate [82]. THADA and HMGA2 genes are studied in GWAS and they are associated with type 2 diabetes mellitus [83,84]. Susceptibility locus of type 1 diabetes mellitus contains RAB5B gene, γAP and ZNF21. Both genes are not associated with ovarian function but are related to apoptotic and proliferative functions of the cell [85]. Table 3 illustrates different single nucleotide polymorphism identified by different GWAS studies in women.

## 9. Specialized Genes Involved in PCOS Pathophysiology

### 9.1. Gene Involved in Ovarian and Adrenal Steroidogenesis

During the evaluation and identification processes, different genes have been found to associate with PCOS hyperandrogenism.

*CYP19* is the gene responsible for aromatase p450 activities, which are required for estrogen formation, and is located in the 15q21.2 chromosome. Lean and obese PCOS patients show lower aromatase activity [86].

The enzyme P450c17α encoded by *CYP17* catalyzes the conversion of pregnenolone to 17-hydroxypregnenolone and progesterone to 17-hydroxyprogesterone. The overexpression of CYP17 in theca cells and polymorphism in the promoter region was found to be associated with PCOS [87,88,89].

The *CYP21* gene is involved in the encoding of an enzyme responsible for conversion of 17-hydroxyprogesterone to 11-deoxycortisol, a step of steroid hormone synthesis. Inactivity of the enzyme leads to ineffective anabolism of steroids, which is further responsible for PCOS [90].

The enzyme involved in rate-limiting step of cholesterol conversion to progesterone is encoded by *CYP11a* [91]. Different studies by scientists have found an association between CYP11a and PCOS as polymorphism and variation has been reported in CYP11a [92,93,94].

### 9.2. Epigenetics of PCOS

Epigenetic changes are responsible for PCOS due to transgenerational and mitotic heritable processes and not due to DNA sequence changes. Different animal models (rat, sheep, and monkey) have reported hyperandrogen production in the fetal state [95]. These animals also showed PCOS-like symptoms. Some clinical studies also reported the same results in offspring when they are predisposed to increased androgens, and they later presented with PCOS-like symptoms [96,97,98]. Chromatin modification (without additions or deletions in pre-existing DNA) helps in epigenetic reprogramming.

Two specific mechanisms are involved in this process.

CPG Island Methylation—methylation, hydro methylation, formylation, or carboxylation at cytosine in its 5th carbon of pyrimidine and guanine ring [99,100].

Gene expression is inhibited by DNA methylation but hydromethylation is involved in the increase in DNA methylation [101].

Histone modification—Acetylation, methylation, ubiquitination, and phosphorylation reactions are involved in epigenetic reprogramming. Somatic and germ cells can both be genetically reprogrammed but generational changes can only be transmitted by germ cells to offspring [99].

Upregulation or downregulation of DNA gene expression may occur when epigenetic alterations take place. These changes further affect translation and protein synthesis. FST (a gene involved in the encoding of follistatin), LMNA (encoding Lamin/AC), EPHX1 (encodes for epoxide hydrolase), and PPARGC1A (encode for peroxisome proliferation are building blocks for physiological processes such as follicular development, insulin, glucose metabolism, inflammation processes, and steroidogenesis. Disturbed genetic methylation results in physiological imbalance, that leads to syndromic conditions [100] (Table 4).

#### 9.2.1. Peripubertal Diet and Epigenetics of PCOS

Diet can affect the methylation status of sex steroids and growth-related genes (CYP19A1, HSF11B2, IGF2), which further influences puberty onset in adolescent girls [102,103]. Makroin ring finger protein 3 (MKRN3) and delta-like noncanonical notch ligand 1 (DLK1) imprinted mutation leads to the precocious onset of puberty. Kisspeptin (KISS1) and its receptors, such as KISS1R mutation among these genes, also results in the advance onset of puberty. KDNy (Kisspeptin/neurokinin B/dynorphin) neuron, located in the arcuate nucleus of the hypothalamus, is involved in the regulation of GnRH release. This neuron can be affected by epigenetic modifications and sirtuins (energy -sensing proteins), which are coupled with NAD+ (metabolic factor). A recently reported study explains that the expulsion of SIRT1 from KISS1 neuron leads to produce-changed chromatin state. The over-nutritional level among adolescent girls was responsible for this condition, which results in the advance onset of puberty [104]. Another study reported in mice described a specific deficient diet (folate, methionine, and choline) in adolescent age resulted in increased plasma levels of (thcy) total homocysteine, which further contributes to important events in the hippocampus, i.e., promotor hypermethylation and loss of expression of the glutamate receptor 1 (Gria1), and produces impaired memory and learning [105]. Epigenetic bases for dietary-mediated puberty onset and mammal’s neuroendocrine influence on ovarian function were supported by these studies [106,107].

#### 9.2.2. Prenatal Diet and Epigenetics of PCOS

Most of the understanding we acquired about the prenatal dietary influence on epigenetics comes from reported different non-human primates, rodents, and sheep studies. This reports the consequential effects of the maternal diet and metabolism on prenatal androgenization [108,109]. The provided treatment exposure for such conditions during early and mid-gestation reflects the key stage for the androgen-sensitive gonadal development [109]. In the ovaries of adult female offspring, genome-wide alterations in DNA methylation indicates a gene network associated with PCOS [108].

### 9.3. Genes Involved in Insulin Action and Secretion

*CAPN10*—Calpain-10 is a protein encoded in humans by the CAPN10 gene. The Calpains are a family of calcium-dependent, cysteine proteases. This gene encodes a large sub-unit. Additionally, Calpain-10 (CAPN10) is an atypical calpain because it lacks the calmodulin-like calcium-binding domain and it instead has a divergent C-terminal domain [110]. This gene is associated with type 2 or non-insulin-dependent diabetes mellitus (NIDDM) and is located within the NIDDM1 region. Multiple alternative transcript variants have been described for this gene. Furthermore, CAPN10 is a protein-coding gene. Diseases associated with CAPN10 include type 1 diabetes mellitus 2 and PCOS. Among its related pathways are integrin pathway and ERK signaling. Gene ontology (GO) annotations related to this gene include cytoskeletal protein binding and calcium-dependent cysteine-type endopeptidase activity. An important paralog of this gene is CAPN3 [111,112].

In PCOS, patients suffer from disturbed insulin levels. As per previous studies, CAPN10 mutation is associated with PCOS [113]. The CAPN10 gene was the first to be identified as a risk gene of type 2 diabetes [114]. Furthermore, the CAPN10 gene consists of multiple single nucleotide polymorphisms. Both UCSNP-63 and UCSNP-19 polymorphism have been found to be associated with PCOS [115]. Another study conducted on Asian women confirms the association of UCSNP-45, UCSNP-19, and UCSNP63 polymorphism as a risk factor for PCOS [116]. Various case-control and meta-analysis studies have been conducted in different study populations to discuss the correlation between CAPN10 polymorphism and metabolic trait of PCOS (Table 5).

*Insulin receptor substrate protein (IRS)*—Binding of insulin to its receptor leads to auto-phosphorylation. Activation of tyrosine kinase further activates and phosphorylates IRS-1 and IRS-2. These activated substrate proteins are utilized for downstream processing. Polymorphisms among genes (IRS1 and IRS2) have been found for insulin resistance. Gly972Arg polymorphism for IRS-1 and Gly1057Asp polymorphism for IRS-2 have shown high susceptibility to diabetes mellitus [121,122]. Initially, there was no difference found between IRS-1 Gly972Arg and IRS-2 Gly1057Asp alleles in PCOS patients and control [123,124,125]. Subsequent studies report patients with PCOS have shown a high frequency of Arg972 polymorphism in IRS-1 [123]. A study reported by (Dilek et al.) found PCOS women with Gly972Arg were more obese, more insulin-resistant, and have high level of fasting insulin as compared to control and other PCOS patients [125]. In a meta-analysis study, IRS1 Gly972Arg is considered as PCOS-susceptible allele and risk factor for which is responsible for increased level of fasting glucose [126,127]. It has been proposed that decreased tyrosine phosphorylation of IRS-1 and increased phosphorylation of IRS-2 Ser 312 are the molecular mechanisms of insulin resistance among PCOS patients [128]. A study suggests hyperandrogenic environment of PCOS is responsible for increased mRNA level of IRS1 and IRS2 [129]; although, insulin receptor gene polymorphism is considered more important for PCOS etiology compared to IRS1 and IRS2 polymorphism.

*Insulin gene*—The ovarian theca cells have receptors involved in androgen production, whereby insulin plays an important role in this process [130]. Furthermore, the Phosphoinositide 3-kinases/protein kinase B pathway is activated in ovarian theca cells of PCOS patients, which is involved in insulin activation for this process [131]. An excess of insulin is associated with high androgen production [132,133]. The insulin gene is a sandwich gene between the insulin growth factor (IGF-II) and tyrosine hydroxylase at 11p15.5 position [134]. Variable number of tandem repeats (VNTR) occupies a 5′ untranslated region [135]. VNTR polymorphism is responsible for the regulation of insulin gene (INS) and IGF-II transcription rate. This VNTR polymorphism is associated with the polycystic ovarian syndrome [136].

*INSR*—The heterotetrametric proteins composed of two alpha and two beta chains are encoded by this gene. Initially, an exact association between INSR and PCOS was not found [137,138]. In this process, a large chromosome part (19p13.2) was isolated and D19S884 was positively associated with PCOS [139]. This part of the chromosome also contains INSR. Various recent studies in different populations suggest that despite the ethnic and race variation among population, a strong association has been found in variety of INSR gene and PCOS. Additionally, INSR could be considered a good genetic marker for PCOS (TABLE). The polymorphism rs2059817 and rs1799817 in INSR gene was most widely associated with insulin resistance among PCOS women in various populations [140,141,142]. The insulin resistance mediated by INSR is produced only in metabolic tissues liver, fibroblast, and skeletal muscle, whereas pituitary tissues and ovaries remain insulin sensitive [143].

### 9.4. Genes Involved in Steroid Hormone Effect

#### 9.4.1. Androgen Receptor Gene

The Androgen receptor gene contains 11 exons and encodes a long tri-domain protein of 90kb; this gene is present in the q arm of chromosome X [144]. PCOS is reported in patients due to chromosome X mutation and cellular structural disruption [15]. It has been shown in different studies that menstrual irregularities, anovulation, and microcyst appearance in the ovaries are due to increased androgen levels [32,145]. Experimental studies also indicated that the intrauterine exposure of androgen leads to PCOS development in adult life [146]. Androgen activity and PCOS prevalence are defined by genetic polymorphism in the AR gene in exon one with CAG repeat [147]. An increased frequency of short AR CAG repeat has been found in different studies, indicating androgen gene’s contribution in PCOS occurrence in Chinese and Caucasian population [148,149]. Androgen receptor upregulation and increased androgen sensitivity are also associated with this polymorphism [150,151]. However, Slovenian, Indian, Croatian, and Korean studies found no association between CAG repeat length and PCOS [152,153,154,155]. A study reported a significant connection of XCI (X chromosome inactivation pattern) with PCOS pathogenesis while comparing PCOS families where sisters paired with XCI diversity have more versatile symptoms of PCOS as compared to sisters paired with identical XCI profile [156]. The XCI pattern can also influence the expression of a gene implicated in preovulatory follicular development BMP15 (Xp11.2) [157]. Increased FSHβ transcription and secretion are associated with BMP15 expression while LH expression is not affected [158]. Another study reported no significant difference between the AR CAG-BM level to DHEAS hormone and AG CAG allele length, XCI pattern, and hirsutism [159]. Chaudhary et al. suggests that the XCI pattern can directly alter the genes required for folliculogenesis (gonadotropins and other genes) by changing LH and FSH levels [160].

#### 9.4.2. Sex Hormone-Binding Globulin Gene

The sex hormone-binding globulin gene (SHBG), which synthesizes a 373 amino acid-containing protein, is present in chromosome 17p13-p12. Androgen (testosterone and estrogen) binding with SHBG protein leads to controlled sex hormone levels in the body [161,162]. Metabolic factors such as androgen and insulin control the synthesis of SHBG by hepatocytes [163,164,165]. PCOS females have a lower level of SHBG, which is an inhibitory effect of hyperinsulinemia on SHBG synthesis [166]. Various studies on PCOS patients have reported that single nucleotide polymorphism in SHBG is associated with this disease [167].

### 9.5. Gene Involved in Gonadotropin

#### 9.5.1. Lutein Hormone and Receptor

The altered level of the luteinizing hormone and its disturbed functions are responsible for androgen excess and anovulation in PCOS [168,169]. Excess luteinizing hormone negatively inhibits follicular-stimulating hormone, which further contributes to decreased estrogen production through androgen transfer, which also stimulates the androgen concentration in ovaries [130]. A study reported a point mutation at (Trp8Arg and IIg15Thr) in luteinizing receptor B sub-unit [170], but this mutation is also reported in 15% non-PCOS women populations, in a study conducted on PCOS women [171].

#### 9.5.2. Follicular Stimulating Hormone Receptor

Mutation in this receptor gene leads to the structural disruption of the encoded protein, thus causing an hormonal imbalance in PCOS women. This gene (situated in the p arm of chromosome 2 containing 14 exons) encodes for a G-protein coupled receptor, which is essential for gonadal development [172,173]. A study reported in North Iraq women showed high-frequency polymorphism in this gene [174].

#### 9.5.3. Anti-Mullerian Hormone

The Anti-Mullerian hormone gene is located in chromosome 19(long arm) at Cytogenetic 13.3, which contains five exons and is involved in encoding a protein responsible for infertility [175]. GWAS and exome-sequencing studies reported different variants in the AMH gene as PCOS disease predictors [15,176]. Two different studies by the same research group identified 37 variants with impaired activity in or near AMH and AMHR2 [177,178]. AMH has been found to decrease CYP17 transcription involved in the encoding of rate-limiting enzyme for androgen production [51,179]. The impaired AMH pathway responsible for hyperandrogenemia in PCOS is probably the decreased AMH signaling and subsequential increase in CYP17 expression [34,180]. An increased level of CYP17 mRNA was found in PCOS theca interna cells as compared to normal reproductive subjects [181]. Ceased follicular transition from primary to secondary stage results in early staged follicles and polycystic ovarian morphology due to AMH, which is further a key characteristic of development PCOS [182,183,184]. The study described the role of the impaired AMH pathway in the pathogenesis of PCOS among patients subgroup [178]. This subgroup population also showed a slightly delayed menarche. The PCOS phenotype can be determined by an impaired AMH pathway mechanism by loss of target gene CYP17 inhibition, which results in an increased CYP17 level and subsequent increase in the testosterone level [178]. Other target genes under AMH regulation are CYP11A, CYP19A, and 3BSHD [185,186].

### 9.6. Other Genes

#### 9.6.1. PCO

This gene is also known as PCOS1, which is present in chromosome 19p13.2. This gene has been studied by several scientists to identify the association. In 1971, this gene was reported in two sisters for the 1st time; furthermore, this study was again replicated in 2005. A large sample of 367 PCOS families were included in the study [139]. As per the study, due to a replication of the results of a previous study by the same scientific group [187], it was concluded that D19S884, a dinucleotide repeat located in the intron of FBN3 (Fibrillin 3) gene 105 bp’3 to exon 55, could be responsible for the transcription of the nearby gene and for INSR. It was also concluded that D19S884 might be involved in post-translational processing of FBN3to mRNA (Table 4).

#### 9.6.2. SRD5A and SRD5B

In 1999, a study was conducted to identify the activity of SRD5A in PCOS women, which was reportedly high in the study [188]. Additionally, 2p23.1 is the cytogenetic location for SRD5B. It was further found that the SRD5A variant is responsible for hirsutism in PCOS patients, while SRD5B is associated with PCOS protection to patients [189].

#### 9.6.3. Fat Mass Obesity (FTO)

This gene, associated with obesity and type 2 diabetics, encodes an enzyme alpha-ketoglutarate located in chromosome 16(q arm). Single nucleotide polymorphism (rs9939609) was associated with PCOS women in Pakistan [190]. This polymorphism was reported to be significantly high in PCOS women compared to the normal population [191].

## 10. Application of Genetic Identification

The genetic identification of any disease is a diagnostic or predictive tool, commonly known as precision medicine. Genetic-risk scores have been used in different studies to discuss the increased variance in diseases and association among diseases and phenotypic comorbidities, type 2 diabetes mellitus disease [192] variance, and coronary heart disease single nucleotide polymorphism-associated myocardial infraction [193]. GWAS in Chinese women have already used this concept to determine genetic loci in PCOS women [194,195] and also in cohort studies reported in English women [196]. Recently, various genetic loci have been identified, which could be incorporated into disease diagnosis, prediction, and treatment.

## 11. Treatment

An ideal treatment approach suited to all PCOS women is not achievable; instead, it provides only symptomatic relief to the patients [197,198]. Due to the complexity of disease (such as infertility, menstrual irregularity, weight reduction, acne, and hirsutism), personalized treatment approaches are required [199,200], depending on the patient’s condition.

### 11.1. Non-Pharmacological Approach

#### 11.1.1. Weight Reduction

PCOS women suffer from obesity, especially in the abdominal area, due to androgen excess. The first treatment approach for such females is to reduce their weight. The study found that 5% weight reduction may restore PCOS women’s menstrual irregularity [201]. Reaching the normal level of BMI results in decreased free testosterone and decreased metabolic syndromes [202].

#### 11.1.2. Diet

A better way to achieve weight reduction is choosing the right food, specifically fiber- rich, low glycemic-indexed, low-saturated fat-indexed food. Additionally, patients should be aware of high glycemic-indexed food in order to avoid them [203].

#### 11.1.3. Exercise

Exercise plays an important role in the weight-reduction process, which is further associated with an improvement in insulin sensitivity [204]. Exercise with or without diet also leads to the regulation of ovulation. The mechanism involved in the ovulatory regulation is the modulation of the hypothalamus–pituitary–gonadal (HPG) axis [205].

#### 11.1.4. Complementary and Alternative Medicines (CAM)

Some studies report that only 60% of PCOS patients treated pharmacologically respond to the therapy [206]. CAM approaches involve nutritional, psychological, physical, and a combination of all these factors [206]. PCOS management has always involved some form of adjuvant therapy, which includes CAM [207]. Moreover, 70% of PCOS females have utilized such treatment once in their treatment process [208]. Traditional Chinese medicine, immunotherapy, yoga, spa, Tai Chi, and diet therapy (herbal, probiotics, and supplementations) are some examples of complementary and alternative medicines [208,209,210,211,212,213].

##### Acupuncture

This alternative therapy has been used in China for more than 3000 years [208]. The thin needles are placed in the skin and muscles for sensory stimulation. The clinical manifestations of PCOS are improved due to increased endorphin production, which increases gonadotrophin-releasing hormone secretion, ovulation, and the menstrual cycle [205,207].

##### Diet Therapy (Health Supplementation)

Along with the medicines, different supplementations have been effective in PCOS females, which include vitamin D, resveratrol, α lipoic acid, omega 3, folic acid, myoinositol, and d-Chiro inositol [208,209,210,211]. Studies have reported that myoinositol, d-chiro inositol taken alone or in combination, results in an increased frequency of ovulation and decreased frequency of FSH-targeted ovulation for pregnancy [212,213,214].

### 11.2. Medicinal Approach

A healthy lifestyle with diet and exercise is always recommended to PCOS-diagnosed patients because patients solely get benefit from diet and exercise in mild to moderate cases [215]. The medicinal approach depends on the patient’s condition and choice. If the patient does not want to get pregnant and reports only menstrual irregularities, combined oral contraceptives (COCs) or progestin are the drugs of choice. Metformin is also a drug of choice, along with COCs, as it restores the ovulatory cycles. If infertility is to be cured, clomiphene citrate or other aromatase inhibitors are treatment options [216].

## 12. Conclusions

PCOS is a complicated disease in terms of its pathophysiology. Its early diagnosis and treatment can be beneficial for patients, as long-term modalities can be delayed or prevented with this approach. The diagnostic approaches for PCOS have progressively enhanced with advancements in genetics. Functional data reported by different epigenetic and GWAS have positively increased our knowledge and understanding of PCOS pathogenesis. The genetic markers have improved diagnosis, which can also distinguish between patients in specific disease phenotypes. An early diagnosis of PCOS with associated comorbidities helps to identify specific treatment for individual patient phenotype, which requires continuous progression in genetic and pathophysiological research.

## 13. Future Remarks

PCOS is a collection of metabolic and reproductive gene variants associated with different biochemical, clinical, and biological features, specifically due to increased androgen production. Females may develop infertility in their reproductive ages; hence, early detection and treatment are required for a better prognosis. Key gene polymorphisms are helpful in the timely diagnosis and screening of PCOS subtype.

An increased number of identified loci for PCOS genetic phenotype indicates the incorporation of genetic testing in disease prediction and diagnosis. PCOS gene mapping provides significant assurance in the identification of susceptible loci, which can further contribute to finding the novel disease pathway and drug targets. Precision medicine is an exciting prospect in which identified genetic risk markers work as a diagnostic tool.

Presently, oral contraceptive pills are the first line treatment for PCOS. These medicines are involved in the correction of menstrual irregularities. A variety other medicines are also included, which may increase the chances of conception. These medicines are associated with different adverse effects related to other pathologies such as cardiac pathology, psychological pathogenesis (poor self-esteem and depression), and diabetes mellitus. To increase the chances of conception, PCOS females are involved in the utilization of more artificial approaches, including IVF, IUI, and laparoscopic drilling.

The advancement in the early diagnostic approaches with genetic variance also potentiates a better prognosis of the PCOS. The early detection of the disease provides a better treatment approach to patients

## Figures and Tables

**Table 1:** Diagnostic Criteria for PCOS.

S.N	Diagnosis Criteria	Symptoms	Recommendations
1.	National Institute of Health (1990)	Biochemical and clinical signs of hyperandrogenism Chronic anovulation	Both criteria are required
2.	Rotterdam Consensus (2003)	Biochemical and clinical signs of hyperandrogenism Oligo and anovulation Polycystic ovaries morphology	Two of three criteria required
3.	Androgen Excess Society (2006)	Biochemical and clinical signs of hyperandrogenism Ovarian dysfunction (oligo anovulation, polycystic ovaries morphology)	Both criteria required

**Table 2:** Phenotypic Difference Based on Diagnostic Criteria. - explains the difference in phenotype symptoms based on diagnostic criteria.

Phenotype	Hyperandrogenism	Chronic Anovulation	Polycystic Ovaries Morphology	NIH 1990	Rotterdam	AE-PCOS 2006
Phenotype A	Yes	Yes	Yes	-	-	-
Phenotype B	Yes	Yes	No	-	-	-
Phenotype C	Yes	No	Yes		-	-
Phenotype D	No	Yes	Yes		-	

**Table 3:** Single Nucleotide Polymorphism Identified by Different GWAS Studies in Women.

S.N	Diagnostic Criteria	Gene Locus	SNPs	Nearest Gene	Study
1.	Rotterdam	2p16.3	rs13405728	LHCGHR STON1-GTF2A1L	Chen et al., 2011 [66]
		2p21	rs12468394		
			rs13429458	THADA	
			rs12478601		
		9q33.3	rs10818854	DENND1A	
			rs10986105		
			rs24779106		
2.	NIH	8p32.1	rs804279	NEIL2, GATA4	Hayes et al., 2015 [63]
		9q22.32	rs10993397	C9orf3	
		11p14.1	rs11031006	ARL14EP, FSHB	
3	Rotterdam	12p12.2	rs10841843	GYS2	Hwang et al., 2012 [68]
			rs6487237		
			rs7485509		
4	NIH	2q.34	rs1351592	ERBB4	Day et at., 2015 [67]
		11q22.1	rs11225154	YAP1	
		2q21	rs7563201	THADA	
		11p14.1	rs11031006	FSHB	
		5q31.1	rs13164856	RAD50	
		12q21.2	rs1275468	KRR1	
5.	Rotterdam	8q24.2	rs10505648	KHDRBS3 LICE02055	Lee et al., 2012 [69]

**Table 4:** Different Genes Involved in Pathogenesis of PCOS.

S.N	Different Gene Categories Involved in PCOS	Genes under Categories
1.	Genes involved in ovarian and adrenal steroidogenesis	CYP19 CYP17 CYP21 CYP11a
2.	Epigenetics of PCOS	NCOR1 PPARG1
3.	Gene involved in insulin action and secretion	CAPN10 IRS-1 IRS-2 INS INS
4.	Gene involved in steroid hormone effect	AR SHBG
5.	Gene involved in gonadotropin	LH AMH FSHR
6.	Other genes	FTO PCO SRD5A SRD5B

**Table 5:** Polymorphism Associated with Calpain 10 gene.

S.N	Gene	Type of Polymorphism	Genetic Marker	Physiological Function	Type of Study with Study Population	Reference
1	C A P N 10	UCSNP-43	UCSNP-43, 19 and 63	Calcium medium, intracellular signaling, insulin secretion	Case control study on Chilean women	[117]
2		UCSNP43	UCSNP-43, 19 and 63		Cross sectional study on Brazilian women	[118]
3		UCSNP44	CAPN10 haplotypes		Haplotype phenotype correlation study on Spanish women	[119]
4		UCSNP-19,45, and 63	SNPs		Meta-analysis on Asian women	[116]
5		UCSNP-19, 43, and 44	UCSNP-19, 43, 44 and 63		Cross sectional study on Spanish women	[112]
6		UCSNP-19,63	UCSNP-19, 63, 43 and 44		Meta-analysis Different population	[115]
7		-	UCSNP-43, rs 3792267		Case control on Indian women	[120]

## Data Availability

Not applicable.
